# Transferable situation recognition system for scenario-independent context-aware surgical assistance systems: a proof of concept

**DOI:** 10.1007/s11548-024-03283-z

**Published:** 2024-11-27

**Authors:** D. Junger, C. Kücherer, B. Hirt, O. Burgert

**Affiliations:** 1 School of Informatics, Research Group Computer Assisted Medicine (CaMed), Reutlingen University, Reutlingen, Germany; 2 Faculty of Medicine, Department of Anatomy, Institute for Clinical Anatomy and Cell Analytics, Eberhard Karls University Tübingen, Tübingen, Germany

**Keywords:** Intraoperative situation recognition, Scenario-independent, Applicability, Transferability

## Abstract

**Purpose:**

Surgical interventions and the intraoperative environment can vary greatly. A system that reliably recognizes the situation in the operating room should therefore be flexibly applicable to different surgical settings. To achieve this, transferability should be focused during system design and development. In this paper, we demonstrated the feasibility of a transferable, scenario-independent situation recognition system (SRS) by the definition and evaluation based on non-functional requirements.

**Methods:**

Based on a high-level concept for a transferable SRS, a proof of concept implementation was demonstrated using scenarios. The architecture was evaluated with a focus on non-functional requirements of compatibility, maintainability, and portability. Moreover, transferability aspects beyond the requirements, such as the effort to cover new scenarios, were discussed in a subsequent argumentative evaluation.

**Results:**

The evaluation demonstrated the development of an SRS that can be applied to various scenarios. Furthermore, the investigation of the transferability to other settings highlighted the system’s characteristics regarding configurability, interchangeability, and expandability. The components can be optimized step by step to realize a versatile and efficient situation recognition that can be easily adapted to different scenarios.

**Conclusion:**

The prototype provides a framework for scenario-independent situation recognition, suggesting greater applicability and transferability to different surgical settings. For the transfer into clinical routine, the system’s modules need to be evolved, further transferability challenges be addressed, and comprehensive scenarios be integrated.

**Supplementary Information:**

The online version contains supplementary material available at 10.1007/s11548-024-03283-z.

## Introduction

Surgical assistance systems support surgeons and the surgical team before, during, and after surgery. If these systems can adapt their functionality based on the environment and situation, e.g., provide filtered clinical information [1] or pending tasks [2], they are defined as being context-aware. The required context awareness can be achieved using data sources already present in the operating room (OR), e.g., endoscope [3] or medical devices [4], or additional sensor systems, e.g., RFID tracker [5]. Existing approaches mostly focus on supporting a specific surgical intervention with defined data sources [6]. As the intraoperative environment changes depending on different factors, e.g., the available data sources or sequence of the surgical intervention between the clinic or actors, solutions need to be adapted to different surgical settings. To reliably recognize contextual information and be aware of the intraoperative situation in different scenarios, transferability should be focused during system design.

Transferability to other scenarios is challenging and many different aspects need to be considered. In the context of intraoperative situation recognition, the main aspects to allow for an applicable and transferable system are: (1) surgical interventions and their variance, (2) surgical environments and their sensors, and (3) surgical situation recognition methods and their implementation. A widely applicable system therefore needs to be flexible on the sensor, recognition, and process layer, thus being able to react appropriately to deviating surgical processes and OR equipment within a scenario. Furthermore, the system needs to be transferable to similar scenarios with known processes or sensors, multiple process and sensor variants, as well as completely new scenarios, e.g., new interventions, sensors, or interpretation methods. Aspects such as configurability, interchangeability, and expandability are particularly relevant to enable applicability for multiple scenarios. The situation recognition system (SRS) of [7] addresses this need and will be the main subject of this work. The system is characterized by a generalized architecture that can be adapted incrementally. By emphasizing transferability beyond specific scenarios, we aim to create a modular framework that not only meets current requirements but also provides a foundation for seamless integration and adaption of scenarios.

## Methods

### Iterative development process

**Fig. 1:**
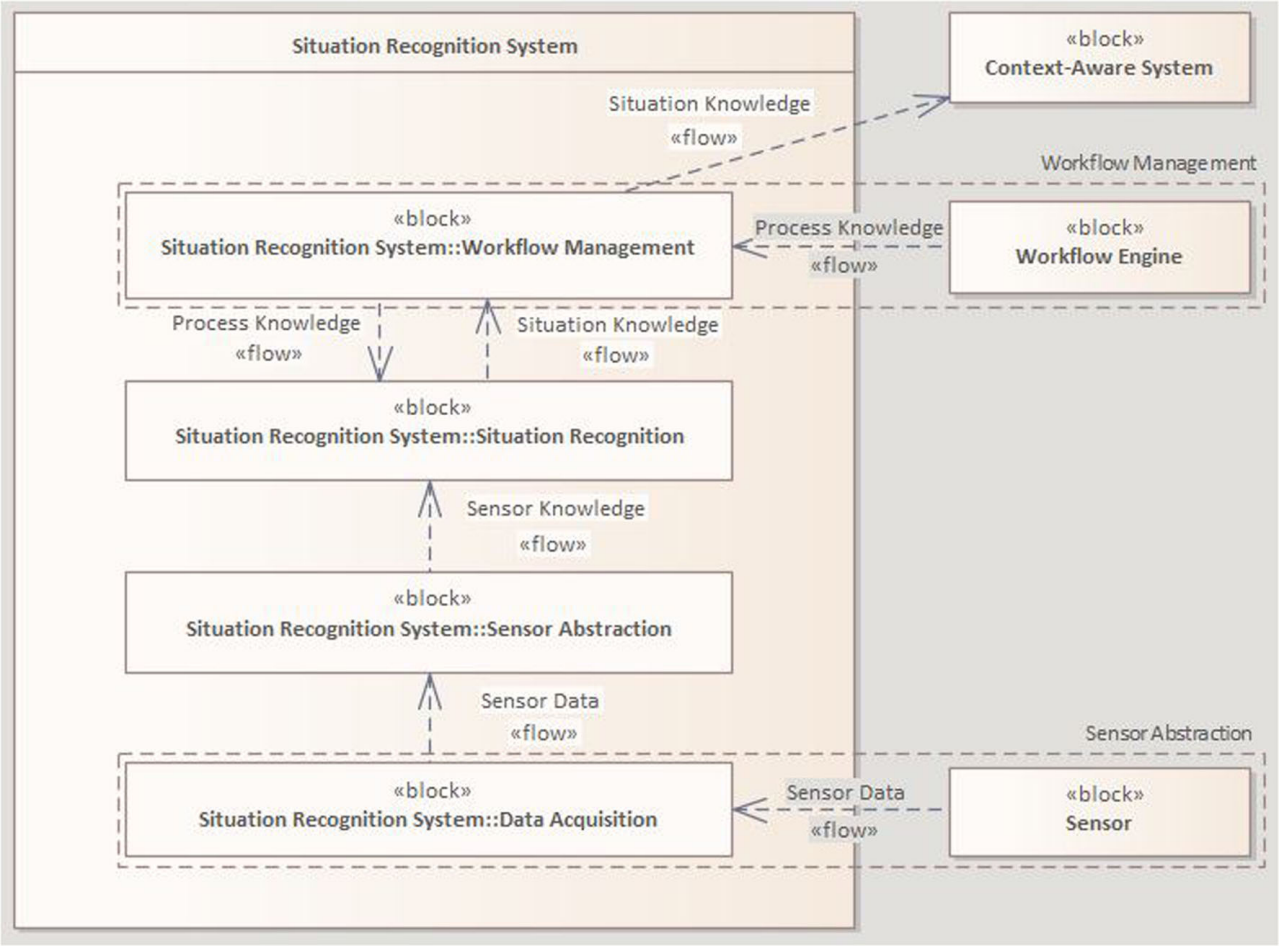
SysML block definition diagram of the components and interfaces of the SRS, condensed representation of the conceptual architecture [7]

We follow an iterative development process, characterized by an incrementally refinement and enhancement throughout the research. Deriving from a state-of-the-art analysis [6], we concluded the need for a flexible SRS in the OR, defined requirements for subsequent development stages, and conducted a high-level concept for a modular system architecture [7]: The SRS shall gather and process data from various intraoperatively available sensors and be aware of knowledge about different interventions. Based on these, contextual information shall be formed and then provided to external context-aware systems (CAS). The concept follows software engineering best practices to realize adaptability and expandability, outlining the structure, components, and interfaces of the envisioned system. It consists of 4 layers: (1) Data Acquisition represents the sensors in the OR, (2) Sensor Abstraction realizes the coupling of sensors and sensor data interpretation, (3) Situation Recognition performs situation interpretation based on sensor and process knowledge, and (4) Workflow Management manages process information using a workflow engine and provides contextual information to CAS (see Fig. 1). Via listener interfaces, the SRS is acquiring *sensor data*. The core of the system is constructed of modules for interpreting the *sensor data* using distributed methods, rules, and machine learning (ML) components. Thereby, *situation knowledge* is retrieved based on the *sensor knowledge,* and *process knowledge* is incorporated via surgical process models depicting the course of surgical interventions. The *situation knowledge* is then provided to CAS. Building upon this concept, a basic framework prototype was developed as a tangible and functional representation of the envisioned system. The initial evaluation demonstrated the overall functionality successfully. In further development cycles, existing features can be refined and new functionalities be integrated via adaptions and extensions (e.g., SDC-based data provision [8] or transferable process models [9]). For more details on the conceptual architecture, please refer to [7].

**Fig. 2:**
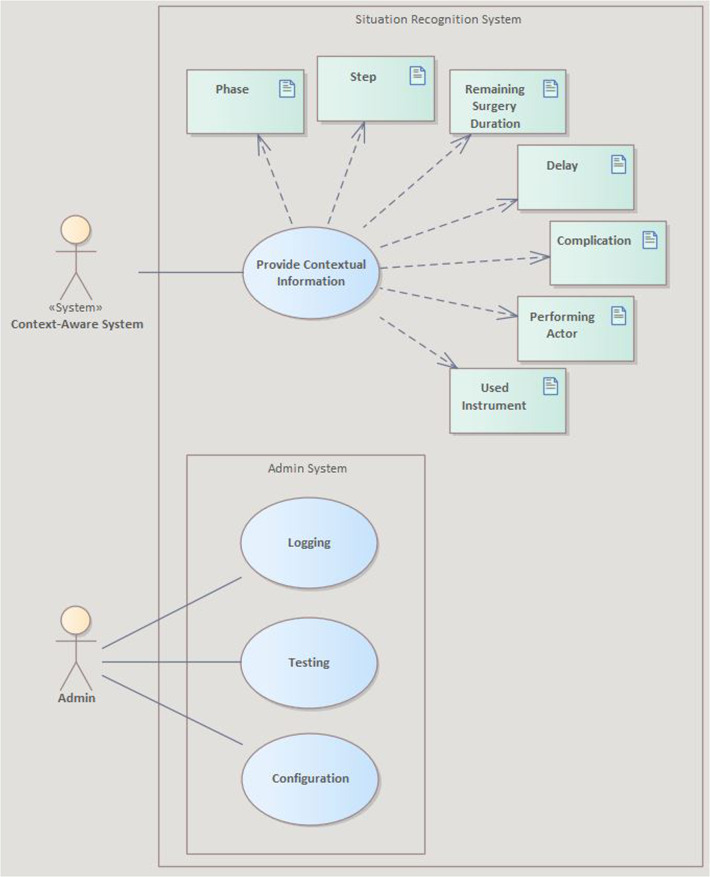
SysML use case diagram of the interactions between users and the SRS

To verify that the SRS is applicable to different surgical settings and transferable to a new context, the evaluation method was specified. We did not find an applicable transferability score in the literature, but the assessment of the quality via non-functional requirements is an established method in software engineering that can be applied to assess transferability. Therefore, we performed a requirements analysis to identify non-functional aspects of the future system according to the ISO/IEC 25010 [10]. This standard defines eight characteristics to categorize product quality properties. The three categories compatibility, maintainability, and portability were stated to have a significant influence on maintenance tasks, therefore being relevant for transferability between scenarios and thus used to refine our requirements. Compatibility defines the degree to which the system and its components can exchange information with other systems or components, maintainability expresses the degree of effectiveness and efficiency with which the system can be modified, and portability describes the degree of effectiveness and efficiency with which the system and its components can be transferred to other environments. Furthermore, we applied the ISO/IEC/IEEE 29148 [11], defining guidelines and characteristics to ensure the quality of requirements. A total of 2 goals and 30 non-functional requirements were derived (see Table 2) for the use case depicted in Fig. 2.

Furthermore, scenarios were integrated to demonstrate the variance of sensors, surgical interventions, and therefore interpretation logic the SRS can support (see Sect. ”Scenarios”). Based on the requirements and three main scenarios, the system was evaluated by the developer (see Sect. ”Functional evaluation”). The overall system was run as a demo prototype for the different sub-scenarios with an automatic sensor data simulation based on realistic data. Aspects that the defined scenarios cannot fully cover were evaluated using additional system tests. For the assessment, the requirements were contrasted to the successful sub-scenario execution as the primary evaluation method. Furthermore, logging details of the interpretation steps and the communication flow as well as code review to obtain further implementation details were used to assess the fulfillment of the requirements. Due to the lack of sufficient data to evaluate aspects like transferability to other clinics, the assessment beyond the requirements is covered by an argumentative evaluation (see Sect. ”Argumentative evaluation”). Thus, a multistage evaluation was realized using requirements, scenarios, and system analysis to assess the ability of the SRS to perform in different and new settings.

### Scenarios

The scenarios listed in Table 1 cover a variety of interventions, sensors, and interpretation logic and serve as the main demonstrators. Each of these covers several sub-scenarios, e.g., by switching between data sources or process models. Further aspects not covered by the three scenarios (e.g., real sensor data via SDC) were evaluated by additional sub-scenarios.

**Table 1:** Overview of the main scenarios of the SRS

	Scenario 1	Scenario 2	Scenario 3
Surgical intervention	Robot-assisted minimally invasive Esophagectomy (RAMIE)	Laparoscopic Cholecystectomy (LC)	Cochlea Implantation (CI)
Processes	Different procedures (step variance)	Different procedures (process variants)	Different procedures (step variance)
Granularities	Phases and steps	Phases	Phases and steps
Process modeling standards	BPMN, CMMN, and combination model	BPMN	BPMN, CMMN, and combination model
Data sources	Instrument and position recognition (simulation), step recognition (checklist)	Phase recognition (simulation of phase flickering), endoscope (dataset), device data (simulation of thermoflator)	Step recognition (checklist and simulation)
Sensor data types	Instrument used and position of surgeon/assistant, name of the step	Name of the phase, endoscope image (instrument used), device parameters of thermoflator	Name of the step
Data formats	SDC and JSON	PNG and JSON	SDC and JSON
Data provision	SDC interface and RESTful listener	RESTful listener	SDC interface and RESTful listener
CAS	*OR-Pad* and CAS simulation (SDC)	CAS simulation (SDC)	CAS simulation (SDC)
Situation data	Phase, step, remaining surgery duration, delay, instrument, and actor	Phase, instrument, and complication	Phase, step, remaining surgery duration, and delay
Interpretation logic basis	Rules	Rules and ML combination	Rules

*Scenario 1* was created based on the cooperation with the University Hospital Heidelberg [12] which provided comprehensive information on process steps, their sequence, and variance, as well as the used instruments and position of the surgeon and assistant in the respective steps. Thereby, data sources of instrument, position, and step recognition could be simulated realistically. Furthermore, rules for situation recognition and process models [9] could be derived. As exemplary CAS, the *OR-Pad* [13] was included. *Scenario 2* was established based on a project of the University Hospital Munich [14] and the publicly available dataset CholecT50 [15]. Different process variants and sensor data, i.e., phase flickering outputs of [14], endoscope image of CholecT50, and exemplarily parameter of a thermoflator of [16], were integrated. In addition, rules were derived, and ML models were trained on different datasets, e.g., CholecT50 and Cholec80 [17]. *Scenario 3* was created in cooperation with the University Hospital Düsseldorf based on the project for an intraoperative checklist [18], which was also used as a data source for step recognition due to the lack of intraoperative sensor data recordings. For the use case, comprehensive information on process steps, their sequence, and variance were provided to adapt process models [9].

## Results

### Functional evaluation

The evaluation resulted in 2 out of 2 completely fulfilled goals and 28 out of 30 fully met non-functional requirements. The results are depicted in Table 2 and are summarized in the following. Details of the assessment are found in the Online Resource 1. Different surgical interventions were simulated based on data from intraoperative sensors and process knowledge (/G01/). The SRS recognizes a variety of contextual information and serves CAS (/G02/).

**Table 2:** Requirements analysis and functional evaluation of the SRS. G = Goal, N = Non-functional requirement

	No.	Goal or Non-functional requirement	Evaluation Result
	/G01/	The SRS recognizes the current situation of different surgical processes in the OR based on data from various intraoperatively available sensors and process knowledge	*Fulfilled*
	/G02/	The SRS provides external systems with contextual information about the current situation of an intervention in the OR	*Fulfilled*
Compatibility	/N01/	The SRS shall connect all external systems (sensors, CAS) through loose coupling	*Fulfilled*
	/N02/	The SRS shall exist in parallel to the OR infrastructure	*Fulfilled*
	/N03/	The SRS shall communicate with the workflow engine via a REST interface	*Fulfilled*
	/N04/	The SRS shall support process models in the modeling standards BPMN and CMMN	*Fulfilled*
	/N05/	The SRS shall communicate with sensors via specified interfaces (e.g., SDC)	*Fulfilled*
	/N06/	The SRS shall be demonstrable with simulated sensors	*Fulfilled*
	/N07/	The SRS shall enable processing data from at least 4 data sources for a scenario	*Fulfilled*
	/N08/	The SRS shall communicate with CAS via an SDC interface	*Fulfilled*
	/N09/	The SRS shall enable to provide data to at least 2 CAS in a scenario	*Fulfilled*
Maintainability	/N10/	The SRS shall enable the exchange of the workflow engine	*Fulfilled*
	/N11/	The SRS shall enable the exchange of process models via the workflow engine for the same intervention	*Fulfilled*
	/N12/	The SRS shall enable the exchange of sensors within a sensor type	*Fulfilled*
	/N13/	The SRS shall enable the exchange of interpretation logic	*Fulfilled*
	/N14/	The SRS shall enable to interpret and provide situation data independently of the CAS currently in use	*Fulfilled*
	/N15/	The SRS shall apply interpretation logic across scenarios if reasonable	*Fulfilled*
	/N16/	The SRS shall enable to import existing process models	*Partly fulfilled*
	/N17/	The SRS shall enable to import existing, trained ML models	*Fulfilled*
	/N18/	The SRS shall provide clear and traceable log entries for information, warnings, and errors	*Fulfilled*
	/N19/	The SRS shall log additional information according to the configured logging level (debug) during administrative use	*Fulfilled*
	/N20/	The SRS shall allow the integration of process models via a workflow engine and knowledge for the intervention via a data management component	*Fulfilled*
	/N21/	The SRS shall allow the connection of sensors via an interface and the adaptation of sensor configurations via a data management component	*Fulfilled*
	/N22/	The SRS shall allow the integration and extension of interpretation logic via modules	*Fulfilled*
	/N23/	The SRS shall offer a GUI for simulating sensor data for testing purposes during administrative use	*Fulfilled*
	/N24/	The SRS shall include a CAS simulation for testing purposes for administrative use	*Fulfilled*
	/N25/	The SRS shall use a test dataset for ML-based approaches for testing purposes during administrative use	*Fulfilled*
Portability	/N26/	The SRS shall enable to add a new scenario, particularly concerning intervention types and sensor types	*Fulfilled*
	/N27/	The SRS shall enable changes to existing process models, connected sensors, and integrated interpretation logic (e.g., ML model) within a scenario	*Fulfilled*
	/N28/	The SRS shall enable the registration and use of available, compatible sensors	*Fulfilled*
	/N29/	The SRS shall allow the configuration of its functionality (connection to the server, intervals, …) via outsourced constants	*Partly fulfilled*
	/N30/	The SRS shall be replaceable by a new instance with a different configuration	*Fulfilled*

#### Compatibility

Different interfaces to sensors are integrated into the SRS, including a RESTful listener, an SDC-based device discovery, and a publish-subscribe SDC interface (/N05/). Sensor data can be simulated via a user interface (/N06/). At least 5 data sources can be used simultaneously, providing single- or multi-sensor data (/N07/). Using SDC interface metrics, several CAS can subscribe to desired information simultaneously (/N08/, /N09/). Sensors and CAS can be coupled and decoupled via listeners and SDC interface, respectively (/N01/). The SRS runs in the research OR with other systems without recognizable restrictions (/N02/). The system communicates with the *Camunda Workflow Engine* [19] via a REST API interface [20] (/N03/). Process models in BPMN, CMMN, and combination models are supported (/N04/).

#### Maintainability

Sensors are assigned to a sensor data type to distribute data to suitable modules of the SRS and can therefore be exchanged (/N12/). Data of every available, configured sensor is automatically incorporated using the system’s implemented listener interfaces (/N21/). Interpretation modules and methods can be exchanged and adapted (/N13/, /N22/). New or modified process models can be integrated via the workflow engine and adaptions to relations, rules, etc. made within data management components (/N11/, /N20/). Due to the REST API interface, the workflow engine can be exchanged, too (/N10/). The SRS continuously interprets knowledge about sensors, processes, and situations (/N14/). The rule- and ML-based interpretation logic is uniformly used for all scenarios but also scenario-specific rules can be defined (/N15/). To reuse established work, trained and tested ML models can be integrated (/N17/, /N25/). Also, process models can be reused but require minimal adjustments for integration (/N16/). To track the system behavior, information is logged in a standardized format at different logging levels, including debug mode (/N18/, /N19/). Furthermore, a GUI for sensor data simulation, defined test cases, and a CAS simulation subscribing to all metrics are available (/N23/, /N24/).

#### Portability

Scenarios can be added and adapted within the SRS (/N26/). Therefore, components and knowledge can be integrated, customized, and exchanged (/N27/). Available, compatible sensors can be registered (/N28/). Outsourced constants (e.g., weightings) allow the configuration of scenarios but no scenario-specific configuration or user interface is provided (/N29/). A new instance can be used with modified settings for the scenarios (/N30/).

### Argumentative evaluation

Because it is impossible to cover all aspects of transferability with test scenarios, we are giving further arguments in this section.

#### Supported scenarios

The scenarios RAMIE, LC, and CI represent realistic demonstrators for the functionality and usability of the prototype, but also other scenarios are supported. Thus, a wide range of variations is covered, offering a versatile platform for demonstrating integrated scenarios. Due to the modular architecture, multiple sensors can be connected, the data are processed in corresponding modules, and different process models and variants can be executed. Switching between supported scenarios requires minimal administrative steps, e.g., to configure the ML model or ensure the connection to sensors and CAS.

#### Effort for new scenarios

The effort for integrating new scenarios or functionalities can vary depending on the complexity and wealth of previously integrated aspects. In the best case, the new scenario is close to an existing scenario, so that the SRS’ functionality can be reused. Otherwise, parts of the functionality have to be modified by adapting or exchanging components. Table 3 shows the main components to identify whether a new scenario can already be supported or adaptions are required. In summary, suitable sensors, interpretation logic, and process information must be available. By integrating further scenarios, the system's complexity will increase, and required adaptations be reduced.

**Table 3:** Components to check required scenario adaptions and extensions

Layer	Aspect	Required, otherwise, be changed
Sensor Abstraction	Sensor and listener	Sensor interfaces
	Data management	Management of sensors (sensor registry)
	Interpretation logic (incl. *Sensor Knowledge*)	Support of sensor (data) type and data format, functionality of modules/methods, coverage of rules and ML models
Situation Recognition	Interpretation logic (incl. *Situation Knowledge*)	Functionality of modules/methods, support of CAS goals
	Data management	Management of intervention information and rules
Workflow Management	Workflow engine and interpretation logic (incl. *Process Knowledge*)	Process models, functionality of methods
	CAS	Functionality of the SDC interface (metrics)

#### Configurability, interchangeability, and expandability

The modular architecture plays a decisive role in coping with changing scenarios. In many cases, configurative adjustments, e.g., change sensors or adapt intervals, can be done by an administrator. In contrast, the integration of new functionalities, e.g., support new sensors, requires to involve system experts to add, modify, or exchange components. Following software engineering best practices, the SRS provides key aspects to achieve adaptability: (1) Encapsulation of functionalities in components, (2) data exchange independent of the internal structure, (3) communication via interfaces with uniform data classes, and (4) central management of constants and general functions. Thus, components can be interchanged and expanded without affecting the whole system.

#### Flexibility of the layers

The scenarios demonstrate that surgical interventions in different variants, a variety of sensor configurations, and different interpretation logic are supported (see Table 1). Thus, the SRS is not limited to a specific scenario but is scenario-independent. Particularly, the usage of generalized and scenario-specific CMMN models [9] gives an outlook on better transferability of process information, whereas the sensor registry and type-specific processing enhance sensor usage on availability. Overall, it nevertheless must be assured for deviating scenarios that depending information and components are covered (see Table 3).

## Discussion

### Applicability and transferability

The scenarios demonstrate the versatility of the architecture, dealing with different data inputs and surgical interventions to react flexibly to other settings. The SRS uses all resources available to generate multi-faceted sensor knowledge. Thereby, generalized and case-specific interpretation logic can principally be used, switched, and combined to deal with the diversity and variety of data. The combination of sensor and process knowledge enables comprehensive situation recognition for different surgical processes. By addressing compatibility, maintainability, and portability, the system is applicable to different integrated scenarios, but also the transfer to deviating situations can be derived. The fulfilled requirements and argumentation highlighted the characteristics to support convenient adaption. For scenarios with similar surgical settings, many of the existing components may be reused to reduce the integration effort. For completely different scenarios, the architecture provides integration support via layers and modules, so that the system can be adapted target-oriented to deviating processes and OR equipment. Concluding, applicability is given as different sensors, logic, and process variants are supported for the integrated scenarios. Moreover, transferability between scenarios is possible due to the easy adaptability for new scenarios and deviating surgical settings. This enables a flexible, transferable situation recognition for different interventions and sensors that adapts to changing requirements and supports widely applicable CAS.

Based on [6], existing approaches are strongly tailored and therefore limited to specific scenarios. Although a few of the existing approaches deal with multiple sensors (e.g., [21, 22]) or show their performance with other datasets (e.g., [23, 24]), the transferability is not shown sufficiently at different levels. In contrast, our SRS was particularly designed to support a variety of data sources and surgical interventions and be easily adaptable, emphasizing features that transcend the scope of specific implementations. This flexibility is possible due to the layers and modules that work independently of the data source and are not limited to a specific surgical domain. While the architecture stays the same, only scenario-relevant components are used in the specific surgical setting. Thus, the SRS offers a platform for different scenarios and addresses increased flexibility and adaptability to further surgical settings. Compared to specified approaches, lower implementation efforts are expected for new scenarios as the architecture already exists, components can be reused, and functionalities are outsourced to enable efficient adaptions and extensions. In summary, the SRS offers a clear advantage over specific approaches due to its architecture, which is geared toward transferability.

### Validity of the results

The validity of the results is assessed according to [25]. *Internal validity* refers to the extent to which valid conclusions can be derived from the evaluation. The prototype was implemented based on the general concept and evaluated by the developer using specific scenarios. Thereby, the input was known and sufficient knowledge of the SRS was available. Expert knowledge may have influenced the assessments of the architecture but is also a prerequisite for adaptions. *External validity* refers to the extent to which the evaluation results can be generalized. The SRS can operate in different scenarios but is not restricted to these. The evaluation highlights the necessary compatibility, maintainability, and portability to realize transferability to other contexts. The realistic scenarios are representative examples, so the results can be transferred to real conditions. *Conclusion validity* refers to the extent to which correct conclusions can be derived from the evaluation. The prototype was evaluated based on requirements, scenarios, and system analysis. Despite the lack of data, the evaluation covers all relevant aspects to assess the SRS’ ability. The transfer to real scenarios and evaluation in a surgical environment is not affected by the actual implementation. *Construct validity* refers to the extent to which the evaluation method can measure theoretical concepts. The chosen evaluation method was multistaged. A functional evaluation based on transferability-relevant requirements and scenarios covering different surgical settings was used to prove the transferability. A complemented argumentative evaluation assessed the transferability beyond the requirements. The chosen approach represents a comprehensive and appropriate procedure for evaluating the SRS.

### Limitations

One of the biggest pitfalls in developing the prototype was the lack of diverse clinical data to cover the information needed at all layers. Although there are selected, freely accessible datasets, these are mainly based on video data (not multimodal data) and, e.g., do not provide detailed information about the surgical intervention process. This lack of available, sufficient data are also stated in other work (e.g., [26–28]) although research is already addressing the representation of greater diversity (e.g., [29, 30]). Hence, the scenarios were realized based on real data from clinical cooperation combined with available datasets to simulate sensor data, implement exemplary interpretation logic, and model clinic-specific process models. Insufficient aspects were enriched exemplary based on observations or logic. As a result, the structure and process chain were realized and a wide range of scenarios were covered. The depicted limitations can be resolved by adapting and expanding the scenarios and functionality in further development cycles. Based on multimodal data, extensive tests would become possible to further investigate the transferability ability, dealing with the diversity and variety of the surgical sensor and process data.

While the prototypical implementation contains specific technical details and limitations, the architectural concept provides the foundation and vision for a flexible and adaptable system that addresses various transferability challenges. Nevertheless, customization or preparatory work will be required before the SRS can be used in a new environment, as the system cannot be capable of everything. It is essential to create specific process models for each new intervention, add interfaces for unknown sensors or, if necessary, train new ML models and define rules to ensure the functionality of the SRS. The depicted SRS can be used in a variety of scenarios and can be adapted to changing requirements with reasonable effort. Compared to systems not designed for transferability, our SRS uses an established modular system design and specifically addresses transferability-relevant requirements engineering characteristics. Nevertheless, as the variability of the necessary adaptations and the lack of comparability do not allow quantifying transferability, the effort can only be predicted as reasonable based on the assessment in this paper.

## Conclusion

Transferability is crucial for versatility and efficiency. The proposed system architecture depicts the approach of a scenario-independent SRS and is characterized by its modularity and adaptability. The framework prototype demonstrates the implementation of the concept, covering a selection of possible sensor variations or interventions. It provides a fundamental platform that, in contrast to scenario-specialized systems, was designed for different settings and can be adapted to new scenarios with expected lower effort. The evaluation method, including a functional and argumentative evaluation, assesses the applicability and transferability of the SRS to different surgical settings. The results show that the architecture is feasible and specifically supports a flexible and transferable SRS that can adapt to environmental changes, can easily be expanded to different surgical settings, and thus enable widely applicable CAS. This proof of concept provides a promising outlook on the possibilities of an SRS that supports many types of intervention and sensor systems and has the required compatibility, maintainability, and portability for new scenarios. In further research, components can be gradually optimized or supplemented to improve interpretation logic and support further scenarios for clinical usage. We assume, that a modular SRS that can be transferred to new scenarios is a key factor in bringing intelligent context-aware surgical assistance systems into clinical routine.

## Supplementary Information

Below is the link to the electronic supplementary material.

Supplementary file1 (DOCX 38 KB)
