# Supplementary material for: Transferable situation recognition system for scenario-independent context-aware surgical assistance systems: a proof of concept
Source: Int J Comput Assist Radiol Surg. 2024 Nov 27;20(3):579–90. doi: 10.1007/s11548-024-03283-z (PMC11929725; doi:10.1007/s11548-024-03283-z)
Supplement: Supplementary file 1 — Supplementary file1 (DOCX 38 KB) [file 11548_2024_3283_MOESM1_ESM.docx]

**Supplementary Material for the Article “Transferable Situation Recognition System for Scenario-Independent Context-Aware Surgical Assistance Systems: A Proof of Concept" of the International Journal of Computer Assisted Radiology and Surgery**

D. Junger^1^, C. Kücherer^1^, B. Hirt^2^, O. Burgert^1^

^1^Reutlingen University, School of Informatics, Research Group Computer Assisted Medicine (CaMed), Reutlingen, Germany

^2^Eberhard Karls University Tübingen, Faculty of Medicine, Department of Anatomy, Institute for Clinical Anatomy and Cell Analytics, Tübingen, Germany

**Corresponding Author**

Denise Junger, ORCID: 0000-0002-7895-3210

Reutlingen University, School of Informatics, Research Group Computer Assisted Medicine (CaMed), Reutlingen, Germany

Contact: denise.junger@reutlingen-university.de, Tel.: +49 (0)7121 – 271 4090

**Online Resource 1: Requirements Analysis & Functional Evaluation**

**Table 1** Requirements Analysis & Functional Evaluation of the Situation Recognition System (SRS). G = Goal, N = Non-functional requirement, CAS = Context-Aware System, OR = Operating Room, SDC = Service-oriented Device Connectivity, ML = Machine Learning, BPMN = Business Process Model and Notation, CMMN = Case Management Model and Notation. The overall system was run as a demo prototype for different sub-scenarios with an automatic sensor data simulation based on realistic data. Aspects that the defined scenarios cannot fully cover were evaluated using additional system tests. For the assessment, the requirements were contrasted to the successful sub-scenario execution as the primary evaluation method. Furthermore, logging details of the interpretation steps and the communication flow as well as code review to obtain further implementation details were used to assess the fulfillment of the requirements. The assessment beyond the requirements is covered by an argumentative evaluation, not included in this resource.

|  | ***No.*** | ***Goal or Non-functional requirement*** | ***Evaluation Result*** | ***Evaluation Assessment*** |
| --- | --- | --- | --- | --- |
|  | **/G01/** | *The SRS recognizes the current situation of different surgical processes in the OR based on data from various intraoperatively available sensors and process knowledge.* | **Fulfilled** | The SRS was demonstrated via the scenarios. Different surgical interventions based on data from various intraoperative sensors and process knowledge were simulated. The SRS recognizes a variety of different contextual information across several levels. |
|  | **/G02/** | *The SRS provides external systems with contextual information about the current situation of an intervention in the OR.* | **Fulfilled** | The SRS was demonstrated via the scenarios. A selection of the recognized contextual information is provided to external systems (CAS). |
| ***Compatibility*** | **/N01/** | *The SRS shall connect all external systems (sensors, CAS) through loose coupling.* | **Fulfilled** | Sensors and CAS can be coupled and decoupled via listeners and SDC interface, respectively. |
|  | **/N02/** | *The SRS shall exist in parallel to the OR infrastructure.* | **Fulfilled** | The SRS runs in the research OR simultaneously with other systems without recognizable restrictions. |
|  | **/N03/** | *The SRS shall communicate with the workflow engine via a REST interface.* | **Fulfilled** | The system communicates with the *Camunda Workflow Engine* via a REST API interface. |
|  | **/N04/** | *The SRS shall support process models in the modeling standards BPMN and CMMN.* | **Fulfilled** | Process models in BPMN, CMMN, and combination models are supported according to the implemented XML parser and are used at runtime. |
|  | **/N05/** | *The SRS shall communicate with sensors via specified interfaces (e.g. SDC).* | **Fulfilled** | Different interfaces to sensors are integrated into the SRS, including a RESTful listener, an SDC-based device discovery, and a publish-subscribe SDC interface. |
|  | **/N06/** | *The SRS shall be demonstrable with simulated sensors.* | **Fulfilled** | Sensor data can be simulated via a user interface and integrated test cases. |
|  | **/N07/** | *The SRS shall enable processing data from at least 4 data sources for a scenario.* | **Fulfilled** | At least 5 data sources can be used simultaneously, providing single- or multi-sensor data. |
|  | **/N08/** | *The SRS shall communicate with CAS via an SDC interface.* | **Fulfilled** | Via the SDC interface, CAS can subscribe to desired information from the SRS via metrics defined in the MDIB. |
|  | **/N09/** | *The SRS shall enable to provide data to at least 2 CAS in a scenario.* | **Fulfilled** | Several CAS can be served simultaneously by the SRS. |
| ***Maintainability*** | **/N10/** | *The SRS shall enable the exchange of the workflow engine.* | **Fulfilled** | Due to the REST API interface, the workflow engine itself can be exchanged. |
|  | **/N11/** | *The SRS shall enable the exchange of process models via the workflow engine for the same intervention.* | **Fulfilled** | A modified process model can be integrated via the *Camunda Workflow Engine* which is then automatically used by the SRS. |
|  | **/N12/** | *The SRS shall enable the exchange of sensors within a sensor type.* | **Fulfilled** | Sensors are assigned to a sensor data type in the data management component (sensor registry) to distribute data to suitable modules of the SRS and can therefore be exchanged with other sensors. |
|  | **/N13/** | *The SRS shall enable the exchange of interpretation logic.* | **Fulfilled** | Interpretation modules and methods can be exchanged and adapted. |
|  | **/N14/** | *The SRS shall enable to interpret and provide situation data independently of the CAS currently in use.* | **Fulfilled** | The SRS continuously interprets knowledge about sensors, processes, and situations. |
|  | **/N15/** | *The SRS shall apply interpretation logic across scenarios if reasonable.* | **Fulfilled** | The rule- and ML-based interpretation logic are uniformly used for all scenarios based on the sensor type and phase/step definition and the configuration, but also scenario-specific rules can be defined. |
|  | **/N16/** | *The SRS shall enable to import existing process models.* | **Partly fulfilled** | Process models (e.g. from other application areas) can be integrated, but require minimal adjustments, e.g. to enable control of the models via the workflow engine. |
|  | **/N17/** | *The SRS shall enable to import existing, trained ML models.* | **Fulfilled** | To reuse established work from other projects, trained and tested ML models can be integrated. |
|  | **/N18/** | *The SRS shall provide clear and traceable log entries for information, warnings, and errors.* | **Fulfilled** | To trace the system behavior, a standardized format on different logging levels (info, warning, error) is used for logging relevant information. |
|  | **/N19/** | *The SRS shall log additional information according to the configured logging level (debug) during administrative use.* | **Fulfilled** | For administrative purposes, additional information on the debug level can be logged. |
|  | **/N20/** | *The SRS shall allow the integration of process models via a workflow engine and knowledge for the intervention via a data management component.* | **Fulfilled** | A new process model can be integrated via the *Camunda Workflow Engine*. Therefore, adaptions to relations, rules, etc. can be made within data management components. |
|  | **/N21/** | *The SRS shall allow the connection of sensors via an interface and the adaptation of sensor configurations via a data management component.* | **Fulfilled** | Data of available, configured sensors is incorporated using listener interfaces. Available sensors need to be configured so that their data can automatically be recorded using the implemented listeners and, if assignable, processed. |
|  | **/N22/** | *The SRS shall allow the integration and extension of interpretation logic via modules.* | **Fulfilled** | Reusable rule- and ML-based approaches can be incorporated. |
|  | **/N23/** | *The SRS shall offer a GUI for simulating sensor data for testing purposes during administrative use.* | **Fulfilled** | A GUI for sensor data simulation and defined test cases can be used for testing purposes. |
|  | **/N24/** | *The SRS shall include a CAS simulation for testing purposes for administrative use.* | **Fulfilled** | CAS simulation subscribing to all metrics provided by the SRS can be used for testing purposes. |
|  | **/N25/** | *The SRS shall use a test data set for ML-based approaches for testing purposes during administrative use.* | **Fulfilled** | The up-to-now integrated ML models use an 80/20-split for training/testing. |
| ***Portability*** | **/N26/** | *The SRS shall enable to add a new scenario, particularly concerning intervention types and sensor types.* | **Fulfilled** | A new scenario can be added and an existing scenario be adapted within the SRS. |
|  | **/N27/** | *The SRS shall enable changes to existing process models, connected sensors, and integrated interpretation logic (e.g. ML model) within a scenario.* | **Fulfilled** | Components and knowledge can be integrated, customized, and exchanged within the SRS. |
|  | **/N28/** | *The SRS shall enable the registration and use of available, compatible sensors.* | **Fulfilled** | Available, compatible sensors can be registered and are then used automatically. |
|  | **/N29/** | *The SRS shall allow the configuration of its functionality (connection to the server, intervals, ...) via outsourced constants.* | **Partly fulfilled** | Outsourced constants (e.g. weightings or intervals) allow the configuration of scenarios, but are bound to the instance of the SRS. No scenario-specific configuration or user interface is provided. |
|  | **/N30/** | *The SRS shall be replaceable by a new instance with a different configuration.* | **Fulfilled** | A new instance can be used with modified settings for all the scenarios. |
